# Supplementary material for: Profiling zero-dose measles-rubella children in Zambia: Insights from the 2024 post-campaign coverage survey
Source: PLOS Glob Public Health. 2025 Dec 30;5(12):e0005265. doi: 10.1371/journal.pgph.0005265 (PMC12753047; doi:10.1371/journal.pgph.0005265)
Supplement: S1 Table — Weighted MR zero-dose prevalence and 95% CIs by age, sex, province, maternal status, household size, and residence. Significant adjusted odds ratios from survey-weighted logistic regression are reported. (DOCX) [file pgph.0005265.s001.docx]

*S1 Table: Zero-Dose Measles-Rubella Prevalence by Demographic and Geographic Characteristics, Zambia, 2024 PCCS*

| **Variable** | **Prevalence (%)** | **95% CI (%)** | **Odds Ratio (95% CI)** |
| --- | --- | --- | --- |
| **Age Group** | | | |
| 0–12 months | 10.50 | 9.32–11.68 | Reference |
| 13–24 months | 12.20 | 10.83–13.57 | — |
| 25–36 months | 13.80 | 12.23–15.37 | — |
| >36 months | 14.99 | 13.25–16.73 | 1.60 (1.27–2.00) |
| **Gender** | | | |
| Male | 11.90 | 10.92–12.88 | — |
| Female | 12.04 | 11.04–13.04 | — |
| **Urban/Rural Status** | | | |
| Rural | 13.02 | 12.04–14.00 | Reference |
| Urban | 10.17 | 9.29–11.05 | 0.76 (0.63–0.92) |
| **Province** | | | |
| Central | 19.15 | 15.84–22.46 | — |
| Western | 17.71 | 15.14–20.28 | — |
| Muchinga | 14.03 | 11.56–16.50 | — |
| Northern | 13.34 | 10.93–15.75 | — |
| North Western | 12.40 | 10.05–14.75 | — |
| Luapula | 11.89 | 9.58–14.20 | — |
| Lusaka | 10.99 | 8.73–13.25 | — |
| Southern | 10.13 | 7.93–12.33 | — |
| Eastern | 9.32 | 7.20–11.44 | — |
| Copperbelt | 6.69 | 4.67–8.71 | — |
| **Maternal Status** | | | |
| Present | 11.00 | 10.06–11.94 | Reference |
| Absent | 19.73 | 15.85–23.61 | 1.74 (1.33–2.27) |
| Deceased | 24.17 | 12.60–35.74 | 2.40 (1.23–4.68) |
| **Household Size** | | | |
| Small (1–4) | 12.29 | 10.72–13.86 | — |
| Medium (5–8) | 11.17 | 9.88–12.46 | — |
| Large (≥9) | 14.18 | 11.63–16.73 | — |

**Note**: Prevalence and 95% confidence intervals (CIs) are weighted estimates from the 2024 Post-Campaign Coverage Survey (PCCS). Odds ratios (ORs) with 95% CIs are shown for significant associations (p < 0.05) in survey-weighted logistic regression, with reference categories as indicated. Dashes (—) indicate no OR calculated due to non-significant association (p > 0.05, e.g., Gender, Household Size) or lack of regression data (e.g., Province). CIs for deceased mothers are wider due to smaller sample sizes.
