# Supplementary material for: Profiling zero-dose measles-rubella children in Zambia: Insights from the 2024 post-campaign coverage survey
Source: PLOS Glob Public Health. 2025 Dec 30;5(12):e0005265. doi: 10.1371/journal.pgph.0005265 (PMC12753047; doi:10.1371/journal.pgph.0005265)
Supplement: S2 Table — Weighted MR zero-dose prevalence and 95% CIs by guardian education, campaign awareness, travel time, reasons for non-receipt, receipt of other antigens, and religion. Significant adjusted odds ratios are shown. (DOCX) [file pgph.0005265.s002.docx]

*S2 Table: Zero-Dose Measles-Rubella Prevalence by Socioeconomic and Access Characteristics, Zambia, 2024 PCCS*

| **Variable** | **Prevalence (%)** | **95% CI (%)** | **Odds Ratio (95% CI)** |
| --- | --- | --- | --- |
| **Guardian Education** | | | |
| None | 12.22 | 10.89–13.55 | — |
| Primary | 11.33 | 9.82–12.84 | — |
| Secondary | 9.40 | 6.32–12.48 | — |
| Higher | 15.53 | 11.75–19.31 | — |
| **Campaign Awareness** | | | |
| Heard | 10.20 | 9.20–11.20 | Reference |
| Not Heard | 18.50 | 16.17–20.83 | 2.00 (1.65–2.42) |
| **Time to Health Facility** | | | |
| <15 min | 9.59 | 8.00–11.18 | Reference |
| 15–30 min | 9.86 | 8.04–11.68 | — |
| 30–60 min | 12.61 | 10.32–14.90 | 1.35 (1.02–1.79) |
| 1–2 hrs | 14.28 | 12.12–16.44 | 1.55 (1.20–2.00) |
| >2 hrs | 25.71 | 10.94–40.48 | 3.20 (1.43–7.16) |
| **Reasons for non-receipt** | | | |
| Lack of awareness | 42.58 | 37.80–47.36 | — |
| Too busy | 19.02 | 14.96–23.08 | — |
| Inconvenient time | 7.69 | 5.20–10.18 | — |
| **Receipt of Other Vaccines** | | | |
| Zero-Dose (No Other Antigens) | 88.75 | 85.81–91.69 | — |
| Received Other Antigens | 11.25 | 8.31–14.19 | 0.26 (0.08–0.91) |
| **Religion** | | | |
| Catholic | 9.95 | 7.62–12.28 | — |
| Protestant | 12.50 | 11.44–13.56 | — |
| Others | 9.84 | 6.74–12.94 | — |

**Note**: Prevalence and 95% confidence intervals (CIs) are weighted estimates from the 2024 Post-Campaign Coverage Survey (PCCS). Odds ratios (ORs) with 95% CIs are shown for significant associations (p < 0.05) in survey-weighted logistic regression, with reference categories as indicated. Dashes (—) indicate no OR calculated due to non-significant association (p > 0.05, e.g., Guardian Education, Religion, Reasons for Non-Receipt) or lack of regression data. CIs for >2 hrs travel time are wider due to smaller sample sizes. Muslim prevalence (0.00%) was excluded due to unreliable estimates from small sample size. Others in Religion includes African Traditional Religion, Non-Religious, and Other Religious Groups.
