# Supplementary material for: Profiling zero-dose measles-rubella children in Zambia: Insights from the 2024 post-campaign coverage survey
Source: PLOS Glob Public Health. 2025 Dec 30;5(12):e0005265. doi: 10.1371/journal.pgph.0005265 (PMC12753047; doi:10.1371/journal.pgph.0005265)
Supplement: Attachment — Submitted filename: Rebuttal Letter.docx [file pgph.0005265.s005.docx]

**Rebuttal Letter**

**Manuscript title:** *Profiling Zero-Dose Measles-Rubella Children in Zambia: Insights from the 2024 Post-Campaign Coverage Survey*
**Journal:** PLOS Global Public Health
**Corresponding author:** Moses Mwale

Dear Editor,

We thank you and the reviewers for the careful evaluation of our manuscript and the constructive comments provided. We have revised the manuscript accordingly and believe that the changes have substantially strengthened the paper. Below, we respond point-by-point to the editor’s and reviewers’ comments and indicate where in the revised manuscript the corresponding changes have been made.

For ease of reference, reviewer comments are reproduced in *italics*, followed by our Response and a brief indication of where changes were made.

1. Editor’s Comments

1.1 Discussion to be more narrative and less bullet-like

*Comment: The Discussion would benefit from a more narrative style and reduced reliance on bullet-like or list-based phrasing. Please revise for flow and cohesion.*

Response:
We appreciate this suggestion. We have rewritten the Discussion in a fully narrative form, grouping key findings and implications into coherent paragraphs and removing any bullet-like structures. The revised Discussion now highlights three main findings, situates Zambia’s burden in the regional context, and links results to implications for routine immunisation (RI) and SIAs in a more integrated way.

Location: Discussion section (paragraphs 1–4).

1.2 Additional references to support recommendations

*Comment: Please add a few recent references to support the recommendations in the policy implications section.*

Response:
We have added several recent references to support our recommendations on health-worker–led communication, mobile/outreach strategies, geospatial microplanning, and digital tools for immunisation system strengthening. These include recent work on mobile phone reminders, mobile teams, high-resolution geospatial mapping, and digital health integration for immunisation.

Location: Discussion – policy implications paragraph (last main paragraph), new references [16–22].

1.3 Data availability and access

*Comment: Ensure that the Data Availability Statement fully complies with journal policy and explains how data can be accessed.*

Response:
We appreciate the comment regarding data availability. The dataset used for this study is owned by the Zambia Ministry of Health and was accessed by the authors strictly under a programme-related data-use agreement that does not permit public sharing of individual-level records. As such, the authors are not legally authorised to deposit the full dataset in a public repository. To support transparency and reproducibility in line with PLOS Global Public Health policy, we have instead provided anonymised summary data and replication outputs as supplementary files. We have also updated the Data Availability Statement to indicate that researchers may request access to the full dataset directly from the Ministry of Health in accordance with national data governance procedures. This approach ensures compliance with ethical and legal requirements while addressing the reviewer’s concern on data accessibility.

Location: Data Availability section (after Acknowledgments).

1.4 Supplementary tables and figures

*Comment: Supplementary tables should be presented as Supporting Information rather than embedded as full detail in the main text.*

Response:
We have retained only the key results in the main text and now refer to Supplementary Tables S1 and S2 for full disaggregated details. Explanatory notes have been adjusted accordingly so that readers are directed to the supplementary material for subgroup estimates and detailed distributions.

Location: Results section (Zero-Dose Prevalence, Demographic Risk Factors, Systemic Vaccination Gaps, and Barriers to Vaccination).

2. Reviewer 1

*Minor revisions are recommended—clarify recall bias mitigation, improve data visualisation, and emphasise data accessibility.*

2.1 Clarify recall bias mitigation

Response:
We agree that further clarification was needed. We have expanded the Data Quality Assurance and Limitations sections to describe how recall bias was mitigated. Specifically, we now note that enumerators were trained in standardised probing techniques, that vaccination status was verified using available home-based records and card photographs, and that event-anchoring was used when cards were not available. We also acknowledge that these measures minimise but do not eliminate recall bias.

Location:

- Methods – Data Quality Assurance (paragraph 2)
- Limitations (first paragraph).

2.2 Improve clarity of data visualisation

Response:
While changes to figure design are primarily reflected in the figure files, we have also enhanced the narrative descriptions of figures to improve clarity. The text now clearly explains what each figure shows (provincial zero-dose prevalence, age and gender patterns, and reasons for non-vaccination) and directs readers to the corresponding supplementary tables for exact values.

Location:

- Results – Zero-Dose Prevalence (Figure 1)
- Results – Demographic Risk Factors (Figure 2)
- Results – Barriers to Vaccination (Figure 3).

2.3 Emphasise data accessibility

Response:
We have strengthened the Data Availability section to explain MoH ownership of the data, the constraints on public sharing of individual-level records, and the steps we have taken to support reproducibility (anonymised summary data and analytical code provided as supplementary material). We also specify how researchers can request access to the full dataset through the Ministry of Health.

Location: Data Availability section.

3. Reviewer 2

1. *Is this a nationwide survey? Please explain.*
2. *Does this survey cover the whole population, or part of the population? What is the percentage of coverage?*
3. *The survey is a prospective study. How does it happen to miss the data? Please explain.*
4. *The statistical part needs more elaboration considering the variables.*
5. *Rewrite the conclusion. Avoid frequency, percent; only mention the findings in relation to the objective.*

3.1 Nationwide nature of the survey

Response:
We thank the reviewer for this important clarification. We now explicitly state that the 2024 MR PCCS was a “cross-sectional nationally representative household survey” conducted across all 10 provinces of Zambia, with stratification by province and urban/rural residence.

Location: Methods – Study Design (first paragraph).

3.2 Population coverage and representativeness

Response:
We have clarified that the survey used a probability-based sample rather than a census of all children. The Sampling Frame and Response Rates sections now explain that the sampling frame was based on the 2022 census, that 370 clusters were selected with probability proportional to size, and that the 8,634 surveyed children, when weighted, represent approximately 2.17 million children aged 9–59 months nationally. We also report the overall household response rate of 97.8%.

Location:

- Methods – Sampling Frame and Sample Design
- Methods – Response Rates.

3.3 Nature of study design and missing data

Response:
We acknowledge the need to clarify this point. We now specify that the PCCS is a cross-sectional post-campaign coverage survey, not a prospective cohort study. We also explain that missing data arose mainly from unavailable vaccination cards, incomplete caregiver recall, and occasional skipped items during interviews, which are typical sources of missingness in household coverage surveys. This is described both in the Data Quality Assurance section and in the Limitations section.

Location:

- Methods – Data Quality Assurance (second paragraph)
- Limitations (first paragraph).

3.4 Expanded description of statistical methods and variables

Response:
In response to this comment, we have substantially elaborated the Statistical Analysis section. We now:

- explicitly describe the use of survey-weighted procedures (stratification, clustering, and weights);
- list the variables included in the analysis by domain (demographic, socioeconomic, geographic/access, and campaign awareness/barrier variables);
- explain the criteria used for selecting variables into the multivariable model (p < 0.10 in bivariable analysis and conceptual importance);
- describe how collinearity was assessed using variance inflation factors (VIF < 3); and
- state how missing data were handled through complete-case analysis given <3% missingness for any covariate.

Location: Methods – Statistical Analysis.

3.5 Revised conclusion (no frequencies; aligned to objectives)

Response:
We have rewritten the Conclusion to remove frequencies and percentages and to present the findings solely in relation to the study objectives. The revised conclusion summarises (i) the persistent burden of MR zero-dose children; (ii) their concentration in specific geographic, age, and household vulnerability groups; and (iii) the structural barriers identified (awareness and distance), and links these clearly to the need to strengthen RI and address access barriers.

Location: Conclusion section.

4. Reviewer 3

*The manuscript is timely and nearly ready; a few editorial issues are suggested for improvement (abbreviations, wording, and consistency across sections).*

4.1 Abstract – background, methods, and conclusion wording

- *Add “(MR)” after Rubella.*
- *Replace “and” with “can” between “communities” and “sustain”.*
- *Insert “was conducted from” before the survey dates and replace the dash with “to”.*
- *Write “routine immunisation” in full with the abbreviation in brackets.*
- *Edit “IA2030” to “Immunization Agenda 2030”.*

4.2 Introduction – abbreviation of RI

*Comment: Insert the abbreviation “RI” in brackets after the first mention of routine immunisation in the main text.*

4.3 Methods – PCCS and MR–SIA abbreviations

*Comment: Use the abbreviations PCCS and MR–SIA consistently after first definition.*

4.4 Results – DPT written in full at first mention

*Comment: Write “DPT” in full the first time it appears and then use the abbreviation.*

4.5 Use of “RI” in Discussion and Conclusion

*Comment: Replace “routine immunisation” with “RI” where appropriate and consistent with earlier usage.*

Responses to Reviewers 3 (4.1 – 5):

We thank the reviewer for the detailed and helpful editorial suggestions. All recommended line-level corrections have been implemented throughout the manuscript. Specifically, we have updated abbreviations and terminology in the Abstract, Introduction, Methods, Results, Discussion, and Conclusion sections, including the addition of “(MR)” after the first mention of Rubella, the clarification of “Immunization Agenda 2030,” and the insertion of full forms before abbreviations such as RI, PCCS, MR-SIA, and Diphtheria–Pertussis–Tetanus (DPT). We also made the suggested wording adjustments in the Methods and Abstract to improve clarity. These revisions enhance consistency, readability, and alignment with standard immunisation terminology. We appreciate the reviewer’s attention to detail and have incorporated all suggested edits accordingly.

Location: Abstract, Introduction, Methods, Results, Discussion and Conclusion.

5. Additional Clarifications and Enhancements

Beyond directly addressing the comments, we have also:

- Clarified the contextual interpretation of COVID-19–related measles outcomes, emphasising that measles vaccination remained strongly protective and that pandemic-era gaps worsened outcomes through reduced coverage rather than any protective effect of being unvaccinated [15].
- Highlighted the three main conceptual contributions of the study: (i) profiling overlap of MR zero-dose with all-antigen zero-dose; (ii) demonstrating the primacy of access and awareness barriers; and (iii) identifying older children and those without maternal caregivers as particularly high-risk groups.
- Ensured consistent UK English spelling (e.g. “immunisation”) throughout the main text, retaining original spelling only in reference titles.

We hope that the revisions meet the expectations of the editor and reviewers. We are grateful for the insightful feedback, which has helped us to improve the clarity, methodological transparency, and policy relevance of the manuscript.

Yours sincerely,

Moses Mwale
On behalf of all co-authors
